# Differential effects of *Calca*-derived peptides in male mice with diet-induced obesity

**DOI:** 10.1371/journal.pone.0180547

**Published:** 2017-06-30

**Authors:** Alexander Bartelt, Anke Jeschke, Brigitte Müller, Isabella Gaziano, Michelle Morales, Timur Yorgan, Timo Heckt, Markus Heine, Robert F. Gagel, Ronald B. Emeson, Michael Amling, Andreas Niemeier, Jörg Heeren, Thorsten Schinke, Johannes Keller

**Affiliations:** 1 Department of Biochemistry and Molecular Cell Biology, University Medical Center Hamburg-Eppendorf, Hamburg, Germany; 2 Department of Orthopedics, University Medical Center Hamburg-Eppendorf, Hamburg, Germany; 3 Department of Osteology and Biomechanics, University Medical Center Hamburg-Eppendorf, Hamburg, Germany; 4 Department of Internal Medicine, Scripps Mercy Hospital, San Diego, California, United States of America; 5 Endocrine Neoplasia and Hormonal Disorders, University of Texas MD Anderson Cancer Center, Houston, Texas, United States of America; 6 Department of Pharmacology, Vanderbilt University School of Medicine, Nashville, Tennessee, United States of America; Universidade do Estado do Rio de Janeiro, BRAZIL

## Abstract

Key metabolic hormones, such as insulin, leptin, and adiponectin, have been studied extensively in obesity, however the pathophysiologic relevance of the calcitonin family of peptides remains unclear. This family includes calcitonin (CT), its precursor procalcitonin (PCT), and alpha calcitonin-gene related peptide (αCGRP), which are all encoded by the gene *Calca*. Here, we studied the role of *Calca*-derived peptides in diet-induced obesity (DIO) by challenging *Calcr*^−/−^ (encoding the calcitonin receptor, CTR), *Calca*^−/−^, and αCGRP^−/−^ mice and their respective littermates with high-fat diet (HFD) feeding for 16 weeks. HFD-induced pathologies were assessed by glucose tolerance, plasma cytokine and lipid markers, expression studies and histology. We found that DIO in mice lacking the CTR resulted in impaired glucose tolerance, features of enhanced nonalcoholic steatohepatitis (NASH) and adipose tissue inflammation compared to wildtype littermates. Furthermore, CTR-deficient mice were characterized by dyslipidemia and elevated HDL levels. In contrast, mice lacking *Calca* were protected from DIO, NASH and adipose tissue inflammation, and displayed improved glucose tolerance. Mice exclusively lacking αCGRP displayed a significantly less improved DIO phenotype compared to *Calca*-deficient mice. In summary, we demonstrate that the CT/CTR axis is involved in regulating plasma cholesterol levels while *Calca*, presumably through PCT, seems to have a detrimental effect in the context of metabolic disease. Our study provides the first comparative analyses of the roles of *Calca*-derived peptides and the CTR in metabolic disease.

## Introduction

It is well established that obesity is linked to components of the metabolic syndrome including insulin resistance, dyslipidemia and a low-grade pro-inflammatory state [1,2,3,4]. Aberrant lipid metabolism in obese adipose and liver tissue is linked to insulin resistance and lipotoxicity. In this context, inflammatory and lipid metabolism gene expression profiles in liver and adipose tissue are strong predictors of metabolic health in humans [5]. Accumulation of excess cholesterol in plasma, in particular as LDL or lipoprotein remnants is linked to the development of atherosclerosis [6]. In the investigation of molecules that may be involved in the regulation of metabolic health in obesity, there is still uncertainty regarding the roles of several circulating hormones such as the calcitonin family of peptides. These include calcitonin (CT) and its precursor procalcitonin (PCT), as well as calcitonin-gene related peptide (αCGRP) all of which are encoded by the *Calca* gene in mice and have been linked to metabolic regulation in mice and humans. Under normal conditions, the primary transcript of the *Calca* gene is subjected to extensive post-transcriptional and post-translational modifications. It is processed into two different mRNAs by alternative splicing, resulting in the synthesis of αCGRP in the central and peripheral nervous system, and PCT in the thyroid gland [7]. Thyroidal PCT is further processed into mature calcitonin (CT) by proteolytic cleavage. Importantly, systemic inflammation as observed in sepsis annihilates tissue specificity and results in ubiquitous *Calca* expression, leading to PCT release from many cell types, including adipocytes and hepatocytes [8,9].

CT is primarily known for its regulatory effects on osteoclast function, while αCGRP was shown to control vascular tone and the activity of bone forming osteoblasts. In contrast, PCT was shown to modulate leukocyte function and survival in experimental sepsis [8,9]. All three peptides derived from the *Calca* gene bind to either the calcitonin receptor (*Calcr*, CTR) or the calcitonin receptor-like receptor (CTRL), two G-protein coupled receptors whose ligand specificity is determined by complexing with three different receptor-activity modifying proteins (RAMPs). Whereas the CTR mediates the biological effects of CT and Amylin (AMY), a peptide co-secreted with insulin and involved in glucose handling, αCGRP and PCT have been shown to exert their biological effects through the CTRL, displaying high levels of expression in the lung and the gastrointestinal tract [10,11]. Despite their pleiotropic effects within the organism, *Calca*-derived peptides have recently been linked to glucose, fat and lipid metabolism: First, salmon CT, exhibiting a much higher pharmacologic potency than mammalian CT, was reported to decrease cholesterol and triglyceride levels, improve energy and glucose homeostasis and attenuate diabetic progression in obese rats [12,13,14]. Second, αCGRP-deficient mice were reported to display increased energy expenditure [15], which is supported by clinical findings showing circulating αCGRP levels to positively correlate with obesity [16,17]. Finally, PCT and αCGRP were both found to be expressed in human adipocytes under various conditions including lipopolysaccharide and glucose-dependent insulinotropic polypeptide stimulation [18,19,20,21]. In this context, a hitherto unknown association of PCT, representing one of the most specific and sensitive markers of bacterial infection and sepsis, with body mass index (BMI), waist circumference, and indices of lipid and glucose metabolism was recently reported [22].

Taken together, these observations point towards a significant role of *Calca*-derived peptides in metabolic regulation. In order to test whether *Calca*-derived peptides are involved in the pathogenesis of obesity and metabolic dysfunction, we performed a comparative study employing three different mouse models that display global CTR-, αCGRP-, or *Calca*-deficiency. These mice were subjected to high-fat diet (HFD) feeding for 16 weeks, followed by the analyses of dynamic glucose tolerance, plasma cytokine and lipid markers, expression studies and histology for assessing metabolic disease.

## Methods

### Mouse studies

*Calca*-, αCGRP- and CTR-deficient mice were generated and genotyped as described previously [23,24,25]. All mice were kept on a C57BL/6 background (backcrossed at least 6 times) and housed in the animal facility of the University Medical Center Hamburg-Eppendorf at 22°C with *ad libitum* access to water and standard laboratory chow diet (Lasvendi). Diet-induced obesity (DIO) was induced in single-caged male mice by feeding a high-fat diet (HFD; Bio-Serv F3282, 35 wt. % lard) *ad libitum*, beginning at 4 weeks of age as described previously [26,27]. For each strain, respective WT (wildtype, C57BL/6 background) littermates were fed at the same time. Standardized necropsies were performed after 4 h fasting around noon. Mice were anesthetized with a lethal dose of Ketamine/Xylazine, blood was withdrawn by cardiac puncture and animals were perfused with PBS (phosphate-buffered saline). Organs were harvested and immediately conserved in TRIzol (Invitrogen), formalin or snap-frozen in liquid N_2_ and stored at -80°C. Throughout all experiments, 8–10 mice were analyzed per group. All experiments were approved by the institutional board at the University Medical Center Hamburg-Eppendorf.

### Plasma parameters

Plasma triglycerides and cholesterol were determined using commercial kits (Roche) that were adapted to microtiter plates. ADM, PCT and CT levels were determined by ELISA (abbexa, Cusabio and Phoenix pharmaceuticals, respectively). For fast performance liquid chromatography (FPLC), pooled plasma was separated using S6-superose columns (GE Healthcare) and lipid levels were analyzed in each fraction as described above. Leptin (R&D), adiponectin (R&D) and insulin (CrystalChem) ELISAs were conducted according to the manufacturer’s instructions. Oral glucose tolerance was assessed after a 4 h fasting period by a gavage of 1 g/kg glucose (Sigma) diluted in 0.9% NaCl (Braun). Blood glucose levels were measured using AccuCheck Aviva sticks (Roche).

### Expression analysis

Tissues in TRIzol^®^ (Invitrogen) were disrupted using a TissueLyser (Qiagen). Total RNA was isolated using NucleoSpin RNA II kit (Macherey & Nagel). Complementary DNA was synthesized using SuperScript^®^ III Reverse Transcriptase (Invitrogen). Quantitative real-time PCR reactions were performed on a 7900HT sequence detection system (Applied Biosystems) using TaqMan Assay-on-Demand primer sets supplied by Applied Biosystems (*Adipoq*: Mm00456425_m1, *Cd68*: Mm03047340_m1, *Emr1*: Mm00802530_m1, *Fasn*: Mm00662319_m1, *Hmgcr*: Mm01282499_m1, *Hmgcs1*: Mm00524111_m1, *Hmgcs2*: Mm00550050_m1, *Il6*: Mm00446190_m1, *Scd1*: Mm00772290_m1, *Srebf2*: Mm01306292_m1, *Tbp*: Mm00446973_m1, *Tnfa*: Mm00443258_m1). Gene expression was calculated as copy number per housekeeper gene TATA box-binding protein (*Tbp*) by the ΔΔCT method and expressed as relative expression to wild-type controls.

### Histology

After sacrifice, mouse organs were fixed in 4% buffered formaldehyde for 24 h, rinsed with PBS, dehydrated in a series of graded ethanol and embedded in paraffin. Sections of 5 μm thickness were cut and stained with haematoxylin and eosin. For immunohistochemistry 5 μm thick sections were cut, dewaxed, microwaved in Target Retrieval Solution (DAKO) for 2 x 4 min and cooled down to room temperature for 40 min. After washing with Tris-buffered saline (TBS), non-specific binding was blocked by incubating sections in 10% normal swine serum (DAKO) for 30 min at room temperature. Slides were incubated with anti-CD68 antibody (ABCAM ab955) at a dilution of 1 μg/ml for 60 min (RT), followed by a biotinylated rabbit anti mouse antibody (DakoCytomation) at a dilution of 1:200 for 30 min. After careful washes in TBS, an incubation with an avidin-alkaline phosphatase complex (ABC kit, Vectastain, Vector) for 30 min followed and thereafter, additional washes in TBS were performed. Alkaline phosphatase activity was visualized using Liquid Permanent Red (LPR) Substrate-Chromogen (DAKO) for 15 min. After washing with water, slides were counterstained with Mayer's hemalum diluted 1:1 in water for ten seconds, blued under water and mounted with Eukitt^®^ (Sigma).

### Liver lipids

For lipid quantification, 50 mg pieces of frozen liver were homogenized in lysis buffer (2 mM CaCl2, 80 mM NaCl, 1% TritonX-100, 50 mM Tris/HCl, pH 8.0). Triglycerides and cholesterol were determined using commercial kits (Roche/Hitachi, Mannheim, Germany). Protein concentrations were measured by a Lowry method, which was modified for lipid containing samples by addition of 0.1% SDS.

### Statistics

Two-tailed, unpaired Student’s T-test was used for comparison of groups except in experiments with multiple groups, which were assessed by one-way ANOVA. *P*<0.05 was considered statistically significant, as indicated by asterisks.

## Results

**Fig 1:**
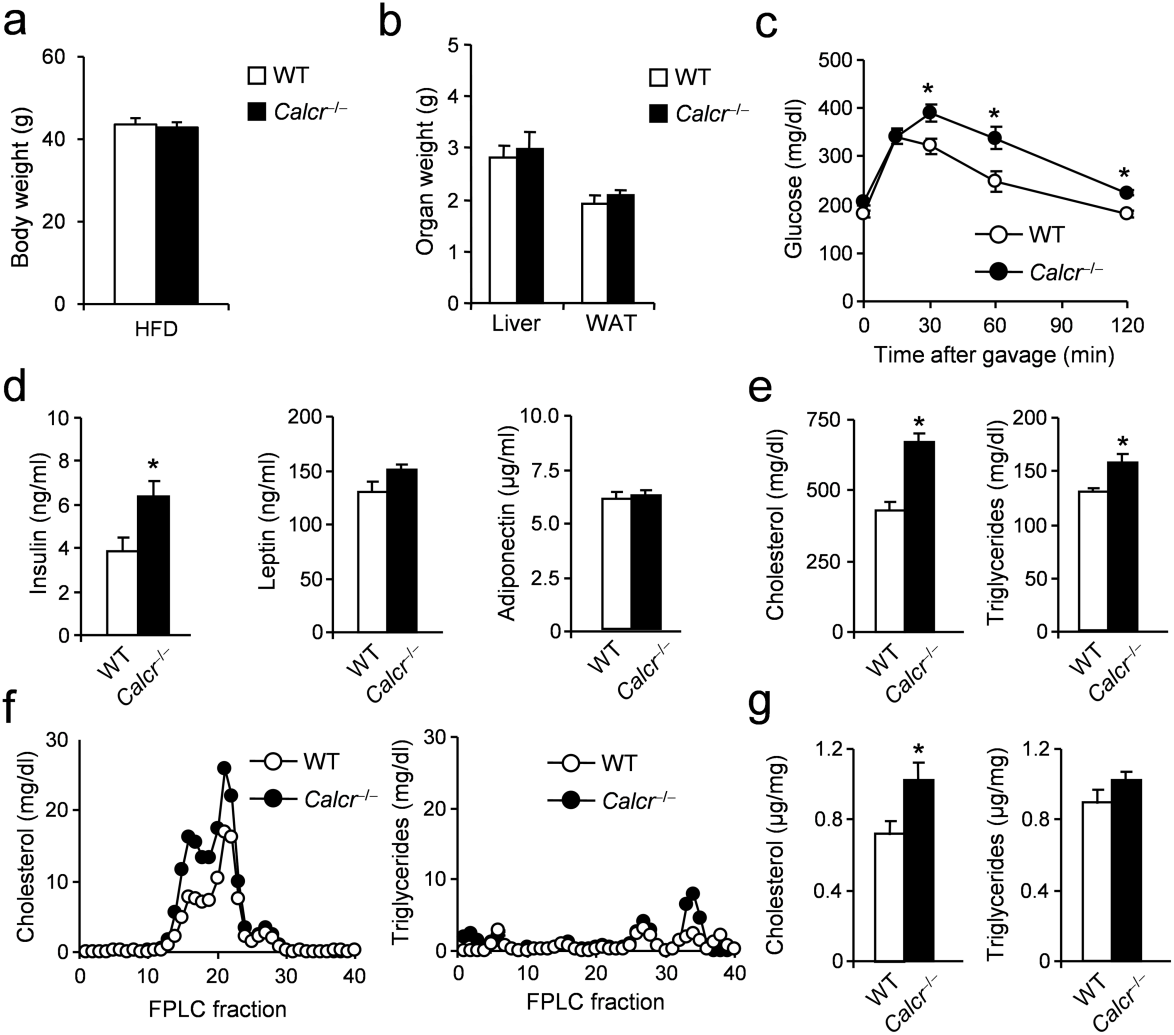
Effects of DIO on metabolic parameters in mice lacking CTR (*Calcr*). (**a**) Body weight in CTR-deficient mice and controls fed HFD for 16 weeks. (**b**) Organ weights (epididymal WAT, white adipose tissue) after 16 weeks of feeding. (**c**) Plasma glucose concentrations during OGTT (1 g/kg) following a 4h fasting period in *CTR*-deficient mice and controls after 16 weeks of feeding. (**d**) Plasma levels of insulin, leptin and adiponectin in the same mice. (**e**) Total plasma cholesterol and triglycerides concentrations in the same mice. (**f**) Cholesterol and triglycerides FPLC profile from pooled plasma (n>8) in CTR-deficient mice and controls fed HFD for 16 weeks. (**g**) Total hepatic cholesterol and triglycerides concentrations in CTR-deficient mice and controls fed HFD for 16 weeks. Results are shown as means ± SEM (*n* = 8–10). *P < 0.05.

In order to study the role of *Calca*-derived peptides in metabolic dysfunction, we first investigated mice lacking the CTR globally. As basic metabolic parameters including body weight, blood glucose, and total cholesterol levels are not altered in CTR-deficient mice under standard conditions [23], we fed CTR-deficient mice and controls a HFD for 16 weeks, starting at the age of 4 weeks in order to induce obesity and associated metabolic disturbances. After the feeding regimen, mice lacking CTR displayed no alteration in final body weight (Fig 1a). Analyses of organ weights at the end of the study revealed no alteration in the weights of liver and epididymal white adipose tissue (WAT) (Fig 1b). Although CTR-deficient mice showed normal glucose levels at baseline, they demonstrated a significantly impaired glucose tolerance beginning 30 minutes after glucose challenge (Fig 1c). The impaired glucose handling was accompanied by increased insulin levels in CTR-deficient animals while no alterations in leptin or adiponectin concentrations were found (Fig 1d). Furthermore, total cholesterol and triglyceride levels were significantly elevated in CTR-deficient animals (Fig 1e). FPLC of plasma samples revealed a peak in the concentration of HDL-cholesterol in CTR-deficient mice, whereas analysis of triglyceride fractions did not reveal any major abnormalities (Fig 1f). To analyze these differences on the tissue level, cholesterol and triglycerides were measured in livers after 16 weeks of feeding. Here, while no alterations in triglyceride concentrations were found, CTR-deficient mice exhibited increased hepatic cholesterol content (Fig 1g).

**Fig 2:**
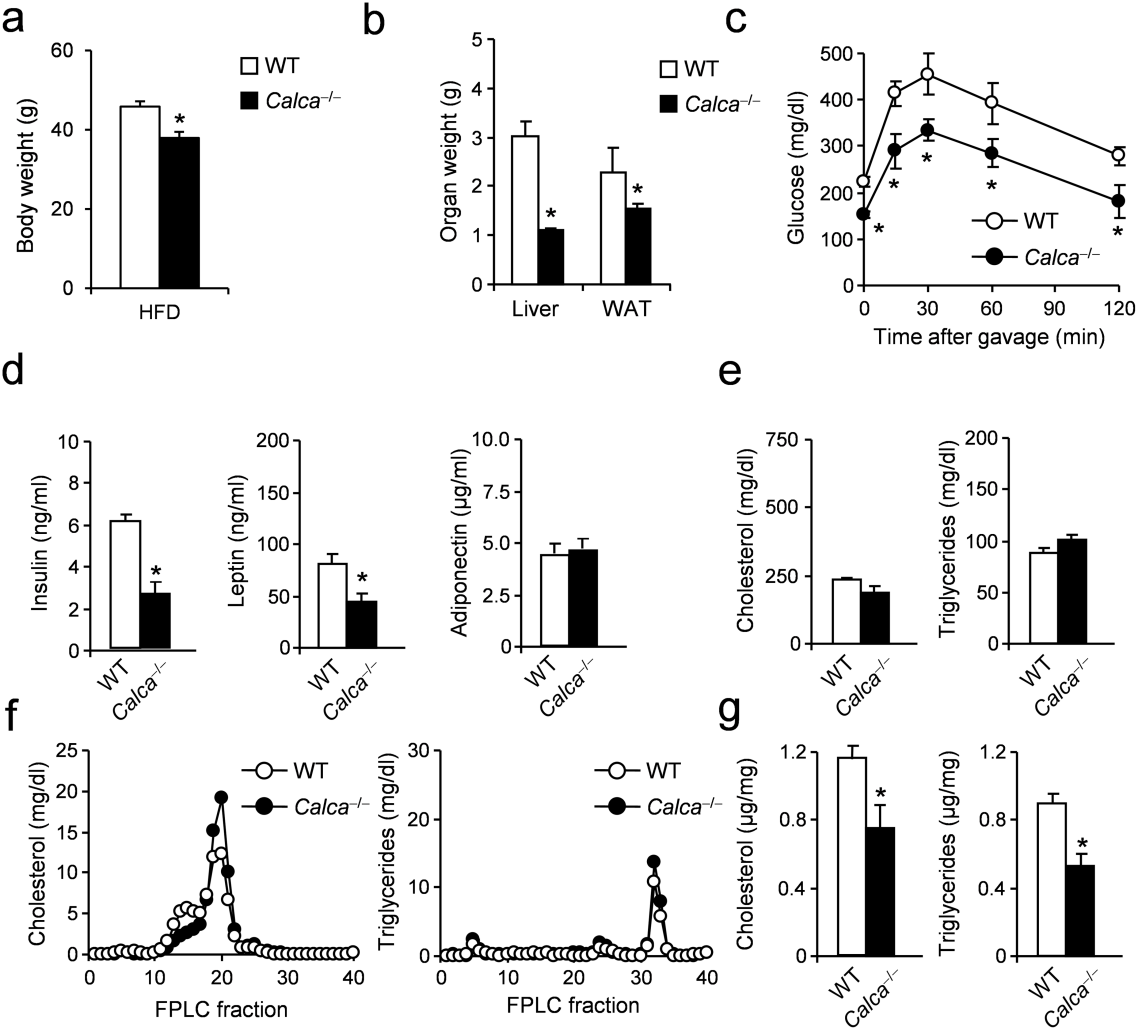
Effects of DIO on metabolic parameters in mice lacking *Calca*. (**a**) Body weight in *Calca*-deficient mice and controls fed HFD for 16 weeks. (**b**) Organ weights (epididymal WAT, white adipose tissue) after 16 weeks of feeding. (**c**) Plasma glucose concentrations during OGTT (1 g/kg) following a 4h fasting period in *Calca*-deficient mice and controls after 16 weeks of feeding. (**d**) Plasma levels of insulin, leptin and adiponectin in the same mice. (**e**) Total plasma cholesterol and triglycerides concentrations in the same mice. (**f**) Cholesterol and triglycerides FPLC profile from pooled plasma (n>8) in *Calca*-deficient mice and controls fed HFD for 16 weeks. (**g**) Total hepatic cholesterol and triglycerides concentrations in *Calca*-deficient mice and controls fed HFD for 16 weeks. Results are shown as means ± SEM (*n* = 8–10). *P < 0.05.

In order to investigate whether these effects can be observed not only in the case of CTR-, but also CT-deficiency, mice lacking *Calca*, encoding CT as well as PCT and αCGRP and exhibiting normal body weights under standard conditions [24], were fed a HFD and analyzed for phenotypic differences following the same experimental protocol as for CTR-deficient mice. At the end of the feeding regimen, *Calca*-deficient mice displayed significantly reduced body weight compared to WT controls, resulting in a weight difference of 7.9 g (WT 45.9 g vs. *Calca* 38.0 g, Fig 2a). Furthermore, a significant reduction in the weights of liver and WAT in *Calca*-deficient mice compared to WT controls was detected at the end of the feeding period (Fig 2b). In sharp contrast to CTR-deficient mice, *Calca*-deficient mice did not only show significantly reduced blood glucose at baseline, they also displayed a significantly improved glucose tolerance at all time points after oral glucose challenge (Fig 2c). Analyses of plasma parameters revealed significantly reduced insulin and leptin levels, while adiponectin levels were unchanged (Fig 2d). Furthermore, total plasma cholesterol and triglyceride levels were unaltered in *Calca*-deficient mice (Fig 2e). Interestingly, the increase in HDL cholesterol observed in CTR-deficient mice was also present in mice lacking *Calca* (Fig 2f). In contrast, hepatic cholesterol and triglyceride content was significantly reduced compared to controls (Fig 2g).

**Fig 3:**
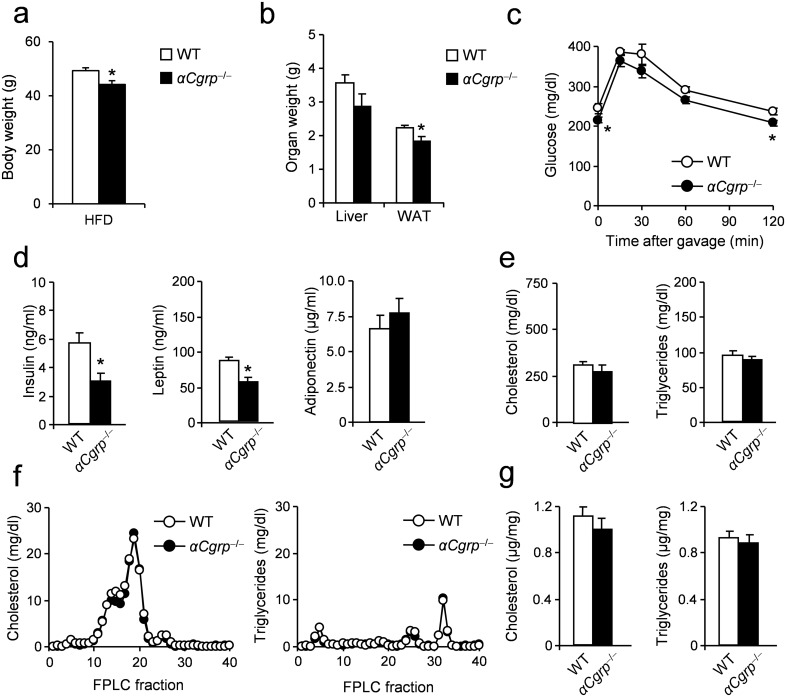
Effects of DIO on metabolic parameters in mice lacking αCGRP. (**a**) Body weight in αCGRP-deficient mice and controls fed HFD for 16 weeks. (**b**) Organ weights (epididymal WAT, white adipose tissue) after 16 weeks of feeding. (**c**) Plasma glucose concentrations during OGTT (1 g/kg) following a 4h fasting period in αCGRP-deficient mice and controls after 16 weeks of feeding. (**d**) Plasma levels of insulin, leptin and adiponectin in the same mice. (**e**) Total plasma cholesterol and triglycerides concentrations in the same mice. (**f**) Cholesterol and triglycerides FPLC profile from pooled plasma (n>8) in αCGRP-deficient mice and controls fed HFD for 16 weeks. (**g**) Total hepatic cholesterol and triglycerides concentrations in αCGRP-deficient mice and controls fed HFD for 16 weeks. Results are shown as means ± SEM (*n* = 8–10). *P < 0.05.

As a previous study suggested a beneficial effect of αCGRP in DIO [15] and a mouse model with exclusive PCT-deficiency is not available to date, we used mice specifically lacking 〈CGRP to investigate any involvement of this peptide in the metabolic phenotype of *Calca*-deficient mice. In this particular mouse model, a stop codon is placed upstream of the 〈CGRP alternative splice transcript, disabling 〈CGRP synthesis yet allowing intact expression of PCT and CT [25]. The respective mice show no alteration in mean body weight under standard conditions [15]. αCGRP-deficiency was associated with a mild however significant reduction in body weight-gain (Fig 3a), similarly to what was reported previously [15]. Moreover, αCGRP-deficient mice displayed only a slight reduction in the weight of WAT, which was not accompanied by any changes in liver weights, as observed in *Calca*- or CTR-deficient animals, respectively (Fig 3b). In contrast to the marked improvement in glucose tolerance observed in *Calca*-deficient mice, αCGRP-deficient mice were characterized by only a slight reduction in blood glucose at baseline and 120 minutes after glucose challenge (Fig 3c). In contrast, αCGRP-deficient mice exhibited similar plasma parameters of glucose metabolism as measured in *Calca*-deficient mice, including decreased levels of insulin and leptin accompanied by unaltered concentrations of adiponectin (Fig 3d). Again differing from *Calca*- and CTR-deficient mice, no changes in HDL levels and plasma or hepatic concentrations of cholesterol and triglycerides were detectable (Fig 3e–3g).

**Fig 4:**
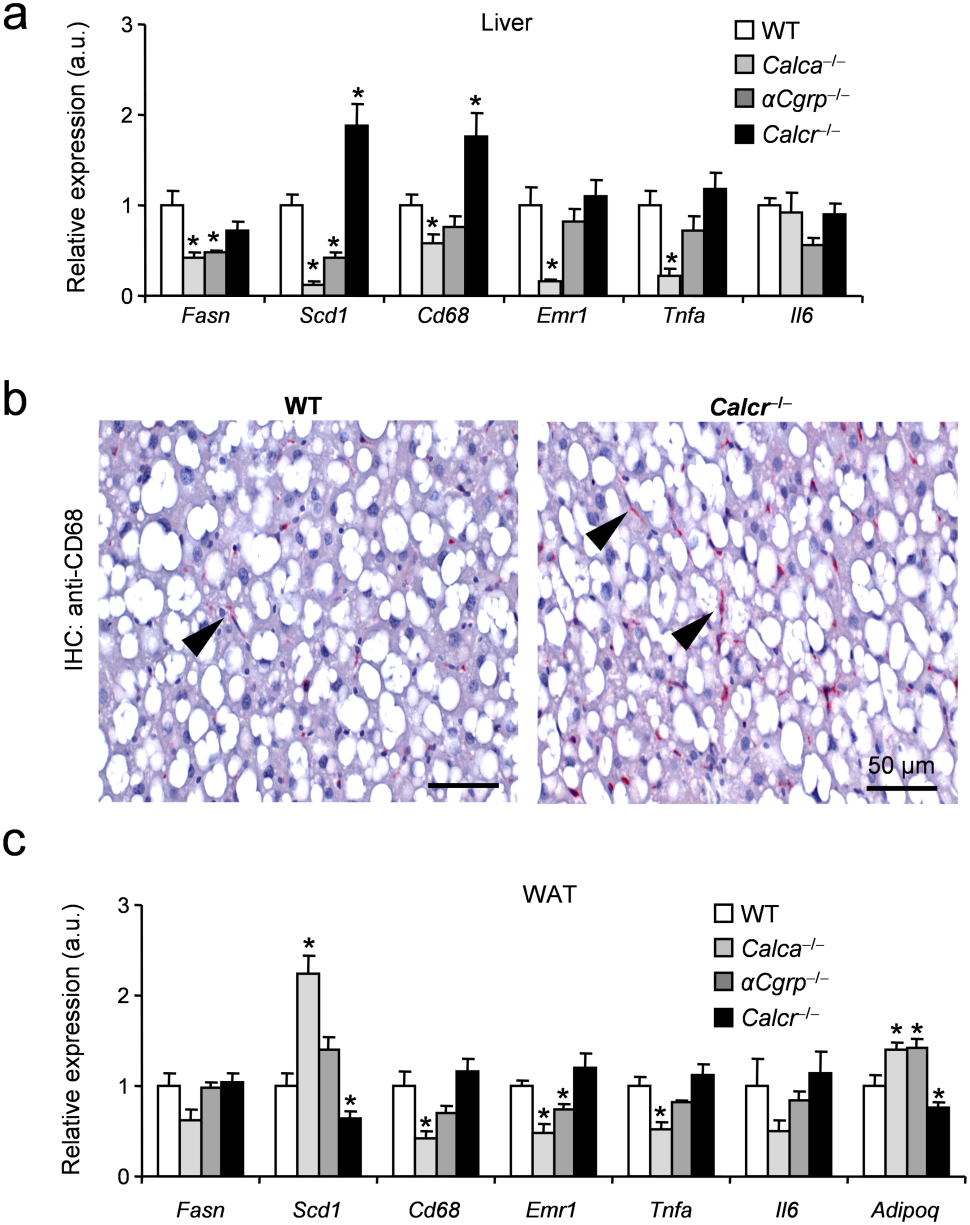
Effects of DIO on the tissue level in mice lacking *Calca*-derived peptides. (**a**) Hepatic expression of selected genes (*Fasn*, fatty acid synthase; *Scd1*, Stearoyl-CoA desaturase-1; *Cd68*, cluster of differentiation 68; *Emr1*, EGF-like module-containing mucin-like hormone receptor-like 1; *Tnfa*, tumor necrosis factor alpha; *Il6*, interleukin 6) of the indicated genotypes after 16 weeks of HFD feeding. (**b**) Representative immunohistochemistry of liver tissue using a Cd68-sepcific monoclonal antibody. Arrows indicate Cd68-positive macrophages. Scale bars 50 m. (**c**) Epididymal WAT expression of selected genes (*Adipoq*, adiponectin) of mice of the indicated genotypes after 16 weeks of HFD feeding. Results are shown as means ± SEM (*n* = 8–10). *P < 0.05.

In order to investigate whether these observations could be explained by molecular differences on the tissue level, we performed gene expression analyses of selected surrogate markers for lipid metabolism and inflammation, which correlate tightly with insulin resistance [27,28] using qRT-PCR after 16 weeks of feeding. Here we found decreased expression of *Fasn*, encoding fatty acid synthase, in the livers of *Calca-* and αCGRP-deficient mice, which was not the case in mice lacking CTR (Fig 4a). In line with this, a significantly decreased hepatic expression of *stearoyl CoA desaturase* (*Scd1*), associated with the metabolic syndrome as well as regulation of inflammation [29,30], was measured in *Calca-* and αCGRP-deficient mice while it was overexpressed in liver tissue derived from CTR-deficient mice. In agreement, the expression of the macrophage markers *Cd68* and *EGF-like module-containing mucin-like hormone receptor-like 1* (*Emr1*) were decreased in *Calca-* and αCGRP-deficient mice and increased in CTR-deficient mice, indicating a more pronounced steatohepatitis in the latter group. Reduced levels of *tumor necrosis factor alpha* (*Tnf*) expression could be identified as a potential mediator of decreased liver inflammation in *Calca*-deficient mice whereas expression of *interleukin-6* (*Il6*) was not altered in any group. To confirm the increased features of NASH in mice lacking *Calcr*, we performed Cd68 immunohistochemistry staining of liver samples and found increased numbers of Cd68-positive macrophages in mice lacking CTR (Fig 4b). The gene expression profiles in liver are usually contrasted in adipose tissue, where markers of lipid metabolism are decreased on the context of obesity-induced metabolic disease [27,28]. Gene expression analyses of WAT demonstrated reduced levels of *Scd1* in CTR-deficient mice, in line with adipose and liver *Scd1* as a strong indicator of metabolic deterioration [28]. In contrast, increased levels of *Scd1* and reduced levels of *Cd68*, *Emr1*, and *Tnf* were measured (Fig 4c), overall indicating limited tissue inflammation and retrograde fatty acid transport in *Calca*-deficient mice. Altogether, our study indicates that loss of CTR has detrimental effects for metabolic homeostasis in the context of obesity and mice lacking *Calca* are protected from HFD-induced weight gain and metabolic deterioration. αCGRP seems to play only a minor role in this scenario, even though, similar to *Calca*-deficient mice, mice lacking αCGRP were characterized by a mild improvement of metabolic parameters after HFD feeding.

## Discussion

The results of the present study provide genetic evidence for a pathophysiologic role of *Calca*-derived peptides in metabolic disease. In particular, we found that genetic inactivation of the CTR in DIO results in impaired glucose tolerance, features of enhanced NASH and adipose tissue inflammation. In addition, we show that the CT/CTR axis is involved in regulating cholesterol levels, and *Calca*, presumably through PCT, may play a deleterious role in metabolic disease.

Although the peptides derived from the *Calca* gene are known for decades, their roles in different physiologic processes and pathologic conditions, in particular in the context of obesity, which is a medical condition reaching worldwide pandemic magnitude, remains unclear. While the roles of CT and αCGRP in bone remodeling and regulation of vascular tone have been studied extensively, their functions in DIO are still ill defined. Furthermore, although representing the most sensitive and specific marker for bacterial sepsis with widespread clinical use, a potential biologic action of PCT remains unclear to date [31,32]. Although we did not detect significant alterations in serum levels of *Calca*-derived peptides during DIO (S1a Fig), this study for the first time provides a comparative analysis regarding the roles of PCT, CT and αCGRP in obesity and metabolic disease using mouse models deficient in *Calca*, CTR, and αCGRP.

With respect to CT, few studies have analyzed its role in DIO so far. This is primarily based on the fact that a suitable mouse model lacking CT signaling has not been available to date. In this study we utilized our recently established mouse model lacking the CTR [23], which does not exhibit the previously reported embryonic lethality of that respective CTR-deficiency model [33,34]. While several recent studies relying on pharmacological approaches demonstrated a beneficial effect of oral salmon CT on body weight, fasting glycaemia and glucose tolerance in rats [12,13,14], our results confirm a potential physiological role of CT in glucose metabolism, as obese CTR-deficient animals displayed features of enhanced NASH, impaired glucose tolerance and hyperinsulinemia *in vivo*. However, while apparently CTR-deficiency did not influence the rate of body weight gain, a more prominent finding was dyslipidemia due to increased cholesterol and triglyceride concentrations, supporting the concept that signaling through the CTR regulates plasma lipid homeostasis independent of weight gain in DIO. As most of the cholesterol change was observed in the HDL fraction it remains to be evaluated how much of this HDL is actually functional or dysfunctional as it has recently been shown that HDL metabolic flux rather than absolute levels determine reverse cholesterol transport [35]. Interestingly, despite the increased cholesterol levels in the liver of CTR-deficient mice we did not observe changes in gene expression of surrogate markers of the SREBP2 cholesterol-sensing pathway [36], neither in the liver nor in WAT (S1b Fig). As we also observed increased plasma lipids and increased hepatic immune infiltrates in CTR-deficient mice, these data may suggest that Kupffer cells and/or other infiltrating immune cells play a role in the pathophysiology in this model. On the other hand, in line with a decrease in body weight on HFD as observed here, mice deficient in *Calca* and αCGRP also showed lower expression of hepatic inflammation markers.

In general our findings partially confirm a recent study demonstrating a beneficial effect of the dual amylin and calcitonin receptor agonist, KBP-089, on weight loss and metabolic parameters in obese rats [37]. Mice lacking the CTR are characterized by increased bone formation and a subsequent increase in circulating osteocalcin [23], an osteoblast-derived peptide increasing secretion of and sensitivity to insulin [38]. While we cannot exclude a potential influence on the metabolic phenotype in our model, a possible beneficial effect of osteocalcin seems to be overridden by the effects of global CTR-deficiency in DIO.

As the CTR not only serves as a receptor for CT, but reportedly also for AMY, CTR-deficient mice additionally serve as a valuable tool to study the role of endogenous AMY in DIO [39]. Although AMY has been implicated in the pathogenesis of diabetes and obesity, leading to the clinical use of its pharmaceutical analogue, pramlintide, as an FDA-approved anti-diabetic drug lowering body weight and hyperglycemia [39], our findings in mice can only confirm CTR signaling role in glucose handling and moreover extend these observations to plasma lipid levels. In particular the effect on HDL levels was pronounced and needs to be explored in more detail towards HDL function and atherosclerosis development. The lack of effect on the rates of body weight gain is in line with previous studies reporting AMY-deficient mice to display only modest or no alteration in body weight [40,41]. The differences between endogenous AMY and its pharmacological actions might be explained by the fact that pramlintide represents a conjunct of human and rat AMY to avoid the highly amyloidogenic effects of the human form. Likewise, it is possible that, apart from the CTR, AMY binds to another hitherto unrecognized receptor other than CTR, as originally suggested by Daquin et al. [33]. In this context, we explored the levels of adrenomedullin, which is thought to bind CTRL but might also act on CTR and exert beneficial effects on HFD-induced metabolic disease [42,43]. However, neither plasma concentrations in lean compared to obese mice, nor gene expression in liver and WAT of CTR-deficient mice showed any differences (S1a and S1b Fig), making it unlikely that adrenomedullin is implicated here.

Based on the receptor pharmacology of the CTR, we additionally monitored the effects of DIO in mice lacking *Calca*. Strikingly, these mice showed a markedly reduced rate of weight gain compared to WT controls, which was in sharp contrast to what we observed in CTR-deficient mice. As *Calca* encodes not only CT, but also PCT and αCGRP, one possibility was that the observed phenotype is caused by the absence of the latter two hormones [7]. To rule out an involvement of αCGRP in the observed phenotype, αCGRP-deficient mice were used as controls. A previous study investigating the role of αCGRP in DIO found αCGRP-deficient mice to exhibit a lower body weight, improved glucose handling and insulin sensitivity which was accompanied by reduced hyperinsulinemia and adiposity compared to controls [15]. Likewise, Riera et al. showed that pharmacologic antagonism of CGRP signaling improved metabolic parameters and potentially inhibits metabolic decline in aged mice (23 months) [44]. In contrast, a recent study demonstrated a beneficial effect of a long acting αCGRP agonist on food intake and body weight in DIO rats [45]. While it is known that pharmacologic agonists may exert different biologic effects, a phenomenon also observed in the case of parathyroid hormone or CT [23] and potentially explaining the findings by Nilsson et al, our study principally confirms the negative effect of endogenous αCGRP on metabolic parameters during DIO. The fact that we observed a less pronounced metabolic phenotype in DIO αCGRP-deficient mice compared to Walker e al. is most likely explained by specific differences in the experimental setup. Walker et al. employed diets with a different fat content 10%, 45%, and 60% compared to 35% in the present study) and fed αCGRP-deficient mice and WT controls for a longer duration (26 weeks of feeding compared to 8 weeks of feeding in the present study). A recent study corroborates the role of αCGRP in obesity as the authors also found that mice lacking αCGRP were partially protected from weight gain on HFD and displayed improved metabolic parameters [46]. In this study, the authors suggest that rather than regulating food intake, αCGRP plays a role in sympathetic output, which warrants further investigation of adaptive thermogenesis and brown adipose tissue activity in this model [47]. As we found αCGRP-deficient mice to exhibit a less pronounced phenotype compared to mice lacking *Calca* with our study protocol, it is reasonable to speculate that the metabolic phenotype of *Calca*-deficient mice is, in part, caused by the absence of PCT, pointing towards a hitherto unknown biologic role of PCT in DIO. This is especially interesting in the context of a recent study demonstrating a positive and significant association of PCT with body mass index (BMI), waist circumference, and indices of lipid and glucose metabolism in humans [22]. Although we cannot provide direct evidence for a detrimental role of PCT in metabolic health, our results may indicate an important impact of PCT signaling during DIO, warranting further mechanistic studies of the underlying molecular pathways.

We are aware of the fact that our study exhibits at least one major limitation. In fact, due to the complex regulation of *Calca* gene expression, we had to use 3 different models including *Calca*-, CTR-, and αCGRP-deficient mice to provide a first comparative analyses on the roles of *Calca*-derived peptides in DIO. Thus, additive or interregulatory effects of the respective peptides cannot be completely excluded, and further studies are required to address the question how the observed phenotypes can be explained mechanistically. Specifically, even though we did not find *Calca*-derived peptides to be regulated in obesity, the individual levels of CT, PCT and αCGRP in the three mouse models studied here might share some mechanistic insight in future. Despite this limitation, our study may prove valuable for future diabetes and atherosclerosis research, as it provides the first comparative characterization of the functions of *Calca*-derived peptides in DIO and associated metabolic disturbances. Our results demonstrate a critical role for CT-CTR signaling for metabolic health. Moreover, these findings point towards a deleterious role of PCT in the pathogenesis of DIO, suggesting PCT as a potential novel molecular target for the treatment of obesity and associated metabolic disorders.

## Supporting information

S1 Fig Systemic and hepatic effects of DIO. (**a**) Serum levels of the indicated peptides in WT mice with DIO (16 weeks of feeding) compared to control fed mice (Adm: adrenomedullin). (**b**) Hepatic and epididymal WAT expression of selected genes (*Srebf2*, Sterol-regulatory element binding factor 2; *Hmgcr*, 3-Hydroxy-3-Methylglutaryl-CoA Reductase; *Hmgcs1*, 3-Hydroxy-3-Methylglutaryl-CoA Synthase 1; *Hmgcs2*, 3-Hydroxy-3-Methylglutaryl-CoA Synthase 2; Adm, Adrenomedullin) of WT and Calcr-deficient mice after 16 weeks of HFD feeding.
(TIF)
