# Supplementary figures and images for: Differential effects of Calca-derived peptides in male mice with diet-induced obesity
Source: PLoS One. 2017 Jun 30;12(6):e0180547. doi: 10.1371/journal.pone.0180547 (PMC5493411; doi:10.1371/journal.pone.0180547)

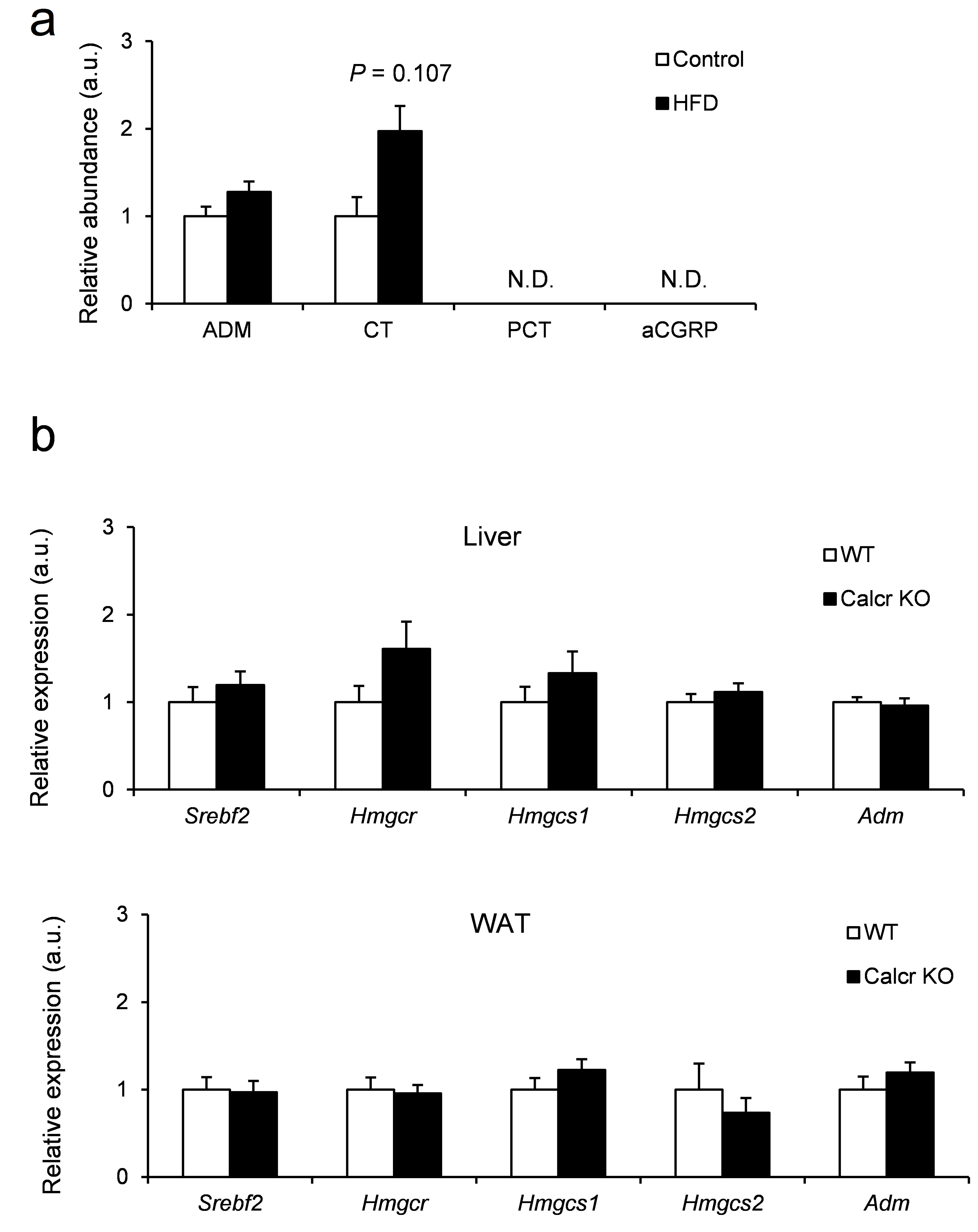

Supplement: S1 Fig — (a) Serum levels of the indicated peptides in WT mice with DIO (16 weeks of feeding) compared to control fed mice (Adm: adrenomedullin). (b) Hepatic and epididymal WAT expression of selected genes (Srebf2, Sterol-regulatory element binding factor 2; Hmgcr, 3-Hydroxy-3-Methylglutaryl-CoA Reductase; Hmgcs1, 3-Hydroxy-3-Methylglutaryl-CoA Synthase 1; Hmgcs2, 3-Hydroxy-3-Methylglutaryl-CoA Synthase 2; Adm, Adrenomedullin) of WT and Calcr-deficient mice after 16 weeks of HFD feeding. (TIF) [file pone.0180547.s001.tif]
